# Evidence Supporting Autotaxin as a Potential New Drug Treatment Target in Patients With Advanced Diabetic Retinopathy

**DOI:** 10.1167/iovs.66.15.49

**Published:** 2025-12-16

**Authors:** Harumasa Yokota, Hiroki Hayashi, Hironori Nakagami, Akifumi Kushiyama, Junya Hanaguri, Megumi Honjo, Makoto Aihara, Makoto Kurano, Koji Igarashi, Sakura Kushiyam, Satoru Yamagami, Taiji Nagaoka

**Affiliations:** 1 Department of Ophthalmology, Asahikawa Medical University, Asahikawa, Japan; 2 Division of Ophthalmology, Department of Visual Sciences, Nihon University School of Medicine, Tokyo, Japan; 3 Department of Health Development and Medicine, Osaka University Graduate School of Medicine, Osaka, Japan; 4 Department of Pharmacotherapy, Meiji Pharmaceutical University, Tokyo, Japan; 5 Department of Ophthalmology, Graduate School of Medicine, The University of Tokyo, Tokyo, Japan; 6 Department of Clinical Laboratory, Graduate School of Medicine, The University of Tokyo, Tokyo, Japan; 7 Department of Clinical Laboratory, The University of Tokyo Hospital, Tokyo, Japan; 8 Bioscience Division, Reagent Development Department, AIA Research Group, TOSOH Corporation, Ayase, Japan; 9 Tokyo Gakugei University, Tokyo, Japan

**Keywords:** autotaxin (ATX), diabetic retinopathy (DR), laser speckle flowgraphy (LSFG), Müller cell, neurovascular coupling, peptide vaccine, proliferative diabetic retinopathy (PDR), rhegmatogenous retinal detachment, type 2 diabetes

## Abstract

**Purpose:**

The purpose of this study was to test the hypothesis that increased intraocular autotaxin (ATX) levels contribute to the progression of diabetic retinopathy (DR), we investigated ATX levels in the vitreous of patients with type 2 diabetes with proliferative diabetic retinopathy (PDR) and assessed the effect of an ATX peptide vaccine (V_A_) in a db/db mouse model of type 2 diabetes.

**Methods:**

Vitreous samples were collected from participants with PDR and from controls with non-diabetic ocular diseases. ATX, lipid mediator, and cytokine levels were measured by ELISA. V_A_ or a control vaccine was administered to db/db mice at 7 and 9 weeks of age. Antibody titers were quantified at 15 weeks of age. Retinal function and blood flow responses to systemic hyperoxia and flicker stimulation were assessed every 2 weeks from age 10 to 14 weeks using electroretinography (ERG) and laser speckle flowgraphy.

**Results:**

Vitreous ATX levels were significantly higher in PDR than in epiretinal membrane or lens dislocation, and positively correlated with ICAM-1, TGF-β1, and adiponectin. V_A_ improved blood glucose levels but not resting blood flow. However, V_A_ restored blood flow responses to both hyperoxia and flicker stimulation and improved ERG implicit times compared with the control vaccine.

**Conclusions:**

Elevated ATX may contribute to the progression of advanced DR. Vaccination against ATX preserved neurovascular function in early diabetic retinopathy in mice. Further studies are needed to clarify its role in DR progression.

Diabetic retinopathy (DR) is a major cause of blindness in the working population.^1^ DR is characterized by vascular abnormalities, typically beginning with microaneurysm formation and retinal hemorrhage. Over a period of years, the disease gradually progresses and leads to the formation of non-perfused areas. If left untreated, DR leads to neovascularization that causes vitreous hemorrhage and tractional retinal detachment. In addition, diabetic macular edema is a form of DR that directly threatens central vision at any stage of DR. Once DR begins to affect vision, normal visual function cannot be restored. Therefore, preventive measures are needed to halt the progression of clinically significant DR.

An imbalance of lipid mediators has been found to play a key role in eye diseases, including DR.^2^ Levels of one lipid mediator, lysophosphatidic acid (LPA), have prognostic value for the development and progression of DR.^2^^,^^3^ LPA is associated with the expression of vascular endothelial growth factor (VEGF) and inflammatory cytokines via binding to specific G-protein-coupled receptors. Thus, inhibiting LPA production is a potential strategy for preventing DR progression.

One approach to reducing LPA production is by inhibiting autotaxin (ATX), a key enzyme that catalyzes the conversion of lysophosphatidylcholine (LPC) into LPA.^4^^–^^6^ Inhibition of ATX might be an effective treatment for DR. In a previous study, researchers compared intraocular levels of ATX in proliferative DR (PDR) and rhegmatogenous retinal detachment (RRD), a condition also accompanied by cell proliferation, and found lower levels of ATX and higher levels of LPA in PDR.^3^ Since then, it has been suggested that intraocular ATX levels are decreased in PDR.^2^ However, it remains unknown how intraocular ATX levels in PDR compare to those in non-diabetic eye diseases without significant cell proliferation. To clarify the role of ATX in PDR, studies should include static retinal diseases, such as epiretinal membrane (ERM), which is not accompanied by neovascularization or significant fibrosis.

In developing a new preventative therapy for DR, it is important to consider long-term efficacy, as patients with diabetes require years-long efforts to prevent disease progression. An ideal preventive treatment should be both effective and durable. For these reasons, vaccine therapy is a promising strategy.^7^ Vaccination for non-infectious diseases, such as hypertension^7^ and Alzheimer's disease,^8^ has been extensively studied.

Therefore, we hypothesized that elevated intraocular ATX may accelerate the pathogenesis of DR through LPA signaling. We measured intraocular levels of ATX in patients with PDR and compared them to those of patients with non-diabetic retinal diseases, including RRD, ERM, and lens dislocation (LD). In addition, we developed a peptide vaccine against ATX (V_A_) and assessed its efficacy in a db/db mouse model of type 2 diabetes to determine its potential as a new strategy for preventing DR.

## Methods

### Collection of Vitreous Samples and Proliferative Membranes

Vitreous samples were collected from patients with PDR and non-diabetic patients with ERM, RRD, and LD between October 2018 and March 2020. The procedures were conducted in accordance with the tenets of the Declaration of Helsinki and were approved by the institutional review board of Nihon University Itabashi Hospital (RK-181211-1). All patients gave written informed consent after we explained the study's purpose and procedures. Undiluted vitreous samples were collected at the start of pars plana vitrectomy using the transconjunctival sutureless vitrectomy system (25G or 27G) and frozen rapidly at −80°C. Proliferative membranes were extracted from patients with PDR with a bimanual technique using a vitreous cutter and forceps. In brief, the surgeon lifted and held the proliferative tissue while cutting it precisely. For patients with ERM, the membrane was visualized with triamcinolone acetonide dispersion and removed with forceps. The tissues were immediately placed in 4% paraformaldehyde (PFA) at 4°C and stored overnight. The extracted membranes were then washed with phosphate buffer saline (PBS) 3 times for 10 minutes each and stored at 4°C until use. Patients with diabetes with a history of intraocular surgery were excluded from the study, except for those who received intravitreal anti-VEGF treatment and retina photocoagulation. The non-diabetic patients had no history of surgery. Patients with RRD had no clinical signs of proliferative vitreoretinopathy (PVR) grade B or worse.

### Measurement of Vitreal ATX Levels

Intravitreal levels of ATX were determined using a 2-site immunoenzymetric assay, as previously described.^9^ The assay reagents were compatible with the AIA system (Tosoh, Tokyo, Japan), a commercial automated immunoassay analyzer that includes automated specimen dispensation, reaction cup incubation, bound/free washing, 4-methylumbelliferyl phosphate substrate dispensation, fluorometric detection, and result reporting.

### Measurement of Cytokines in the Vitreous

Intravitreal levels of cytokines were determined by enzyme-linked immunosorbent assay. Briefly, 50 µL vitreous sample per subject was applied to Human Luminex Discovery Assay/Magnetic bead-based multiplex assay for the Luminex platform (R&D Systems, Minneapolis, MN, USA). The procedures were performed according to the manufacturer's instructions.

### Measurement of Vitreal Lysophospholipids Using Liquid-Chromatography Tandem Mass Spectrometry

Intravitreal levels of lysophospholipids were quantified as previously described.^10^ Briefly, 10 µL vitreous sample was used for identification and quantification of lysophospholipids. Samples were mixed with a 10-fold volume of methanol and an internal standard and then sonicated. After centrifugation at 21,500×*g*, the resulting supernatant was recovered and used for liquid-chromatography mass spectrometry (LC-MS) analyses. Eleven acyl chains (14:0, 16:0, 16:1, 18:0, 18:1, 18:2, 18:3, 20:3, 20:4, 20:5, and 22:6) were monitored and the total LPC and LPA were calculated.

### Immunohistochemistry

The tissues were stained for ATX (1:100, #07-1420; Sigma-Aldrich, St. Louis, MO, USA) and CD31 (1:100, #ab119341; Abcam, Cambridge, MA, USA) overnight at 4°C. The following day, they were incubated with secondary donkey anti-rabbit IgG (H+L) Alexa Fluor 488 (1:400; Thermo Fisher Scientific, Waltham, MA, USA) for 2 hours at room temperature. Immunofluorescent images were obtained using a BZ-9000 microscope (Keyence, Osaka, Japan).

### Animal Preparation

The animal experiments were conducted in accordance with the Ethical Committees of Nihon University Faculty of Pharmacy Committee Guidelines for the Care of Laboratory Animals and the principles of the Association of Research in Vision and Ophthalmology.

One week before the experiment, we purchased 7-week-old male C57BL/6J (*n* = 24) and 5-week-old male C57BL/KsJ-db/db mice (BKS.Cg-Dock7^m^ +/+ Lepr^db^/J; *n* = 12) from Charles River Laboratories Japan, Inc. (Yokohama, Japan). The mice were housed in a temperature-controlled room with a 12-hour light/dark cycle and free access to food and water in a specific pathogen-free condition. The sample size was calculated using EZR, assuming a mean difference of 40 between the 2 groups, a common standard deviation of 20, a significance level of 0.05, and a power of 0.8. It was determined that a minimum of six animals was required per group.

The animals were anesthetized with inhaled 2% isoflurane (Pfizer Japan Inc., Tokyo, Japan) at a constant flow rate of 1.5 L/min throughout the experiment. A heated blanket maintained rectal temperature at 37°C to 38°C. Pupils were dilated with 0.5% tropicamide (Santen Pharmaceutical Co., Osaka, Japan). Blood glucose concentrations were measured from the tail vein with a glucose assay kit (Abbott Laboratories, Abbott Park, IL, USA).

### Vaccine Preparation

**Figure 1.**
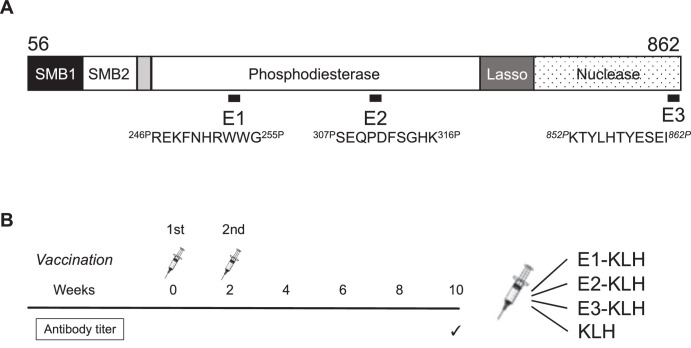
Vaccine preparation. (**A**) The three antigens were selected based on the tertiary structure of autotaxin. E1 contains the binding site to lysophosphatidylcholine. E2 and E3 contain the catalytic site. (**B**) The vaccination schedule for determining the appropriate antigen. Vaccination was performed twice in 2 weeks. Four groups were prepared (E1-KLH, E2-KLH, E3-KLH, and KLH) to compare the antibody titer. KLH, keyhole limpet hemocyanin.

We selected three regions of ATX as antigens based on its tertiary structure. E1 (^246P^REKFNHRWWG^255P^) contains the substrate-binding site, and E2 (^307P^SEQPDFSGHK^316P^) and E3 (^852P^KTYLHTYESEI^862P^) contain enzymatically catalytic areas (Fig. 1A). These antigens were conjugated to keyhole limpet hemocyanin (KLH). For each injection, we prepared 100 µL of peptide vaccine solution, which contained 20 µg of peptide vaccine diluted in 50 µL of normal saline and 50 µL of adjuvant. We used Freund's complete adjuvant (Wako Pure Chemical Industries, Ltd., Osaka, Japan) in the first vaccine and Freund's incomplete adjuvant (Wako Pure Chemical Industries, Ltd.) in the second one.

### Vaccine Optimization

We injected C57BL/6 mice with E1 conjugated to KLH (E1-KLH), E2-KLH, and E3-KLH, and as a control vaccine, unconjugated KLH. Vaccination was performed twice, at 8 and 10 weeks of age, via injection at the nape of the neck. Ten weeks after the first vaccination, 10 µL of blood was collected from the tail vein.

### Antibody Titer Measurement by Enzyme Linked Immuno-Sorbent Assay 

Antibody titer was determined by ELISA as previously described.^11^ Briefly, 10 mg/mL of BSA-conjugated epitope (E1, E2, or E3) was coated on 96-well plate, and incubated at 4°C overnight. After wells were blocked with PBS containing 5% skim milk for 2 hours at room temperature (RT), the diluted sera from 50 to 156,250-fold dilution was added to the wells, and incubated at 4°C overnight. The wells were washed with PBS containing 0.05% tween 20 (PBS-T), and incubated with HRP-conjugated anti-IgG antibody (GE Heathcare) for 3 hours at RT. The washed well with PBS-T were developed the peroxidase chromogenic substrate 3,3′-5,5′-tetramethyl benzidine (TMB; Sigma) for 30 minutes at RT. The colometric reaction was halted by adding sulfuric acid (0.5N). The absorbance of wells were immediately measured at 450 nm using a microplate reader (Bio-Rad). The half-maximum antibody titer of each serum was determined from the highest absorbance in the dilution range (GraphPad Prism 8 software).

### Western Blotting

Two-hundred ng of his-tagged recombinant ATX (R&D systems), 200 ng of BSA-conjugated E2 or E2 antigen were electrophoresed by 4-20% sodium dodecyl sulfate polyacrylamide gel electrophoresis and blotted onto polyvinylidene difluoride membrane (Millipore, Bedford, MA, USA). The blotted membranes were inculcated with sera from mice immunized with E1-KLH, E2-KLH, or KLH (1:500) or with commercially available anti-His antibody (dilution, 1:1,000,000). After subsequent incubation with horseradish peroxidase-conjugated secondary antibody (GE Heathcare), immunoreactivity was detected with Chemi-Lumi One L (Nacalai Tesque). The chemiluminescent signal was detected using a ChemiDoc Touch imaging system (Bio-Rad).

### Vaccination to Test the Effect of V_A_ in db/db Mice

Db/db mice were immunized at 7 and 9 weeks of age, and blood glucose levels, blood pressure (BP), body weight, and antibody titers were measured at 15 weeks of age. Blood glucose levels were measured from the tail vein (TERUMO Co., Ltd., Tokyo, Japan), and BP was measured using the tail-cuff method with a BP monitor (THC31; Softron, Tokyo, Japan).

### Intraocular Pressure and Systemic BP Measurements

Systemic BP and IOP were measured 30 minutes after induction of anesthesia. BP was measured at the tail with an automatic sphygmomanometer (THC-31; Softron). IOP was measured using a handheld tonometer (TonolabTV02; ME Technical, Tokyo, Japan). Mean arterial BP (MABP) was derived from systolic BP (SBP) and diastolic BP (DBP) using the following standard formula: MABP = DBP + (SBP − DBP)/3. The mice were positioned prone during the experiments, and, therefore, ocular perfusion pressure (OPP) was calculated using the following formula: OPP = MABP − IOP.

### Measurement of Retinal Blood Flow 

Retinal blood flow (RBF) was measured with the laser speckle flowgraphy (LSFG)-micro system (Softcare Co., Ltd., Fukutsu, Japan), which is designed for small animals.^12^ The LSFG-micro system uses the same principle as the LSFG system, which has been used to quantitatively estimate ocular (optic nerve head [ONH], choroid, and retina) blood flow in humans and animals. The principle of the LSFG system has been described elsewhere.^13^ Briefly, the coherent laser interacts with moving blood cells to produce backscattered light in a blurred speckle pattern, from which the system generates a mean blur rate (MBR). The MBR obtained from the vascular area of the ONH reflects total retinal circulation and can be used as an index of RBF. The LSFG analyzer software (version 3.2.19.0, Softcare Co., Ltd.) was used to analyze the average vessel MBR. Changes in MBR in response to hyperoxia and flicker light stimulation were expressed as a percentage change from baseline.

### Induction of Hyperoxia

A baseline measurement was obtained before induction of hyperoxia by averaging 3 consecutive flow measurements obtained at 1-minute intervals over 3 minutes. Systemic hyperoxia was induced in the mice via 10-minute inhalation of 100% oxygen, as described previously.^12^^,^^14^ RBF was measured every minutes for 20 minutes, including 10 minutes of stimulation during hyperoxia and 10 minutes of recovery after hyperoxia termination.

### Induction of Flicker Stimulation

The mice were dark-adapted for 2 hours in ambient light reduced to ≤1 lux. Baseline RBF was determined by averaging 3 consecutive flow measurements obtained at 20-second intervals over 1 minute before flicker light stimulation started. Flicker light stimulation was applied for 3 minutes at an intensity of 30 lux, which is appropriate for the rod-dominant mouse retina; 12-Hz flicker stimulation was used, as it has been shown to elicit a maximal RBF response.^12^ RBF was measured at 20-second intervals for 6 minutes, including 3 minutes of stimulation and 3 minutes of recovery.

### Measurement of RBF in Response to Systemic Hyperoxia and Flicker Stimulation

Longitudinal assessment of RBF was conducted in each animal on 2 consecutive days every 2 weeks from 8 to 14 weeks of age (response to systemic hyperoxia was measured on day 1, and response to flicker light stimulation was measured on day 2). An independent masked observer (author A.K.) performed all data calculations and analyses.

We did not measure BP, IOP, or OPP responses because we previously confirmed that systemic BP, IOP, and OPP are unaffected by hyperoxia or flicker light stimulation in mice.^12^

### Statistical Analysis

Intravitreal levels of ATX and cytokines were expressed as median (interquartile range). The Kruskall-Wallis test, followed by post hoc analysis with the Tukey test, was performed to detect a significant difference among the disease groups. For the animal experiments, values were expressed as mean ± standard error. Changes in RBF were calculated as a percentage change from baseline. One-way or 2-way repeated measures analysis of variance (ANOVA), followed by Dunnett's test or the Holm–Sidak test, respectively, was used to detect significant differences across time points within and between groups, as appropriate (EZR and Prism 9; GraphPad Software, San Diego, CA, USA). A *P* value < 0.05 was considered statistically significant.

## Results

### ATX Levels in the Vitreous and Proliferative Tissue in Patients With PDR 

The clinical characteristics of 188 Japanese patients are shown in the Table. In the RRD and PDR groups, participants were predominantly male and younger than the LD and ERM groups. Hypertension was more prevalent in the LD and PDR groups. Hyperlipidemia was also more prevalent in the PDR group. In the PDR group, mean HbA1c was 7.5 ± 1.8%, and mean disease duration was 13.0 ± 10.8 years.

**Table.** Characteristics of Patients

	Total	LD	ERM	RRD	PDR	*P* Value
No. patients	188	9	65	35	79	NA
Sex, M:F	123:65	4:5	34:31	26:9	59:20	0.011
Age, mean (SD) y	62.0 (12.8)	73.9 (14.9)	69.6 (9.2)	59.3 (10.3)	55.6 (12.1)	<0.001
HT, *n* (%)	79 (42.0)	5 (55.6)	22 (33.8)	8 (22.9)	44 (55.7)	0.002
HL, *n* (%)	47 (25.0)	0 (0.0)	15 (23.1)	5 (14.3)	27 (34.2)	0.03
DM, *n* (%)	79 (42.0)	0 (0.0)	0 (0.0)	0 (0.0)	79 (100.0)	<0.001
HbA1c, mean (SD) %		NA	NA	NA	7.5 (1.8)	NA
Duration, mean (SD) y		NA	NA	NA	13.0 (10.8)	NA

ERM, epiretinal membrane; LD, lens dislocation; PDR, proliferative diabetic retinopathy; RRD, rhegmatogenous retinal detachment.

**Figure 2.**
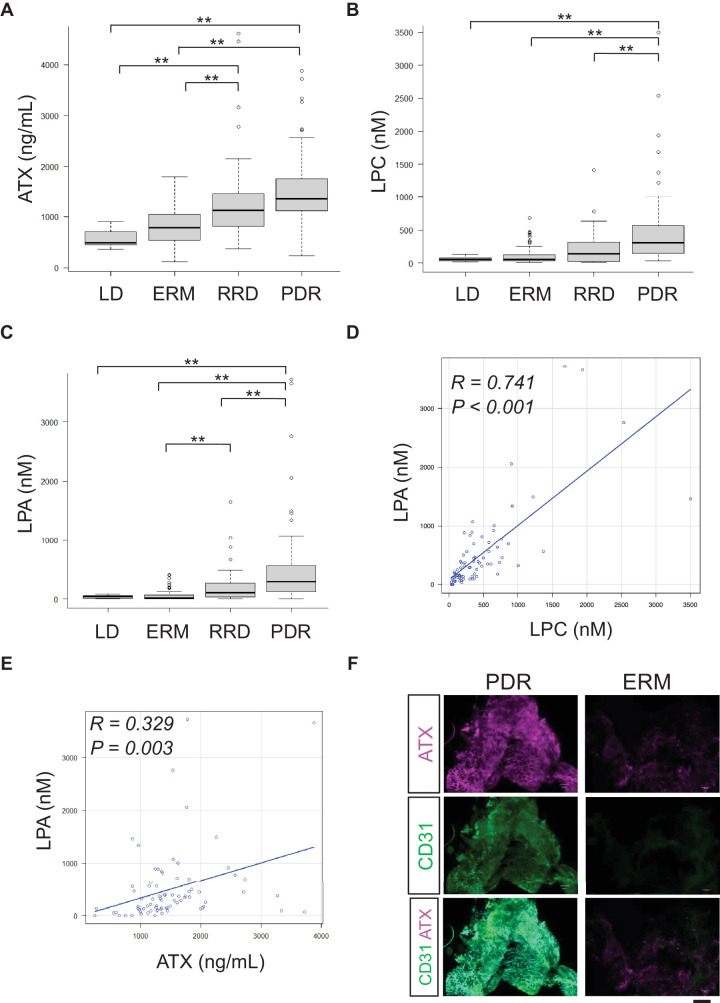
The expression of lipid mediators in the vitreous of participants who underwent vitrectomy. The intravitreal levels of autotaxin (ATX) (**A**), lysophosphatidylcholine (LPC) (**B**), and lysophosphatidic acid (LPA) (**C**) were compared among patients with lens dislocation, epiretinal membrane (ERM), rhegmatogenous retinal detachment, and proliferative diabetic retinopathy (PDR). (**D**) Correlation between LPC and LPA in the vitreous of patients with PDR. (**E**) Correlation between ATX and LPA in the vitreous of patients with PDR. (**F**) Immunohistochemical analysis of ATX expression on proliferative membrane in PDR and ERM. **P < 0.01. *Scale bar* = 200 µm. CD31, cluster of differentiation 31; ERM, epiretinal membrane; LD, lens dislocation; MH, macular hole; PDR, proliferative diabetic retinopathy; RRD, rhegmatogenous retinal detachment.

Intravitreal levels of ATX were significantly higher in patients with PDR (median [interquartile range]: 1351.9 [1119.4–1754.4] ng/mL) than non-diabetic patients with ERM (795.4 [537.7–1040.2] ng/mL) and LD (526.7 [452.8–708.4] ng/mL; *P* < 0.001; Fig. 2A). However, intravitreal levels of ATX in patients with RRD (1133 [831.9–1570.8] ng/mL) did not significantly differ from those in patients with PDR.

We also compared intravitreal levels of LPC and LPA between PDR and non-diabetic retinal diseases (Figs. 2B, 2C). These levels were significantly higher in PDR than in other retinal diseases, including RRD (*P* < 0.001). In patients with PDR, LPA levels were strongly correlated with LPC levels (*R* = 0.741, *P* < 0.001) and significantly correlated with ATX levels (*R* = 0.329, *P* = 0.003).

To further investigate whether ATX is involved in the formation of proliferative tissue in PDR, we performed immunohistochemistry using extracted membranes from the eyes of patients with PDR and non-diabetic ERM (Fig. 2F). In PDR, ATX was strongly co-expressed with CD31-positive cells in proliferative tissue.

### Correlation of Vitreal ATX and Cytokine Levels in Patients With PDR 

Intravitreal levels of VEGF, placental growth factor (PlGF), matrix metalloproteinase-9 (MMP-9), intercellular adhesion molecule-1 (ICAM-1), transforming growth factor (TGF)-β1, and adiponectin were significantly higher in PDR than in non-diabetic diseases in PDR (Figs. 3A–F). Thus, we evaluated the correlation between vitreal ATX and these cytokines (Figs. 3G–M). Levels of vitreal ATX were significantly positively correlated with levels of ICAM-1 (*R* = 0.342, *P* = 0.002), TGF-β1 (*R* = 0.450, *P* < 0.001), and adiponectin (*R* = 0.555, *P* < 0.001). However, no correlation was found between ATX and VEGF, PlGF, or MMP-9 levels.

**Figure 3.**
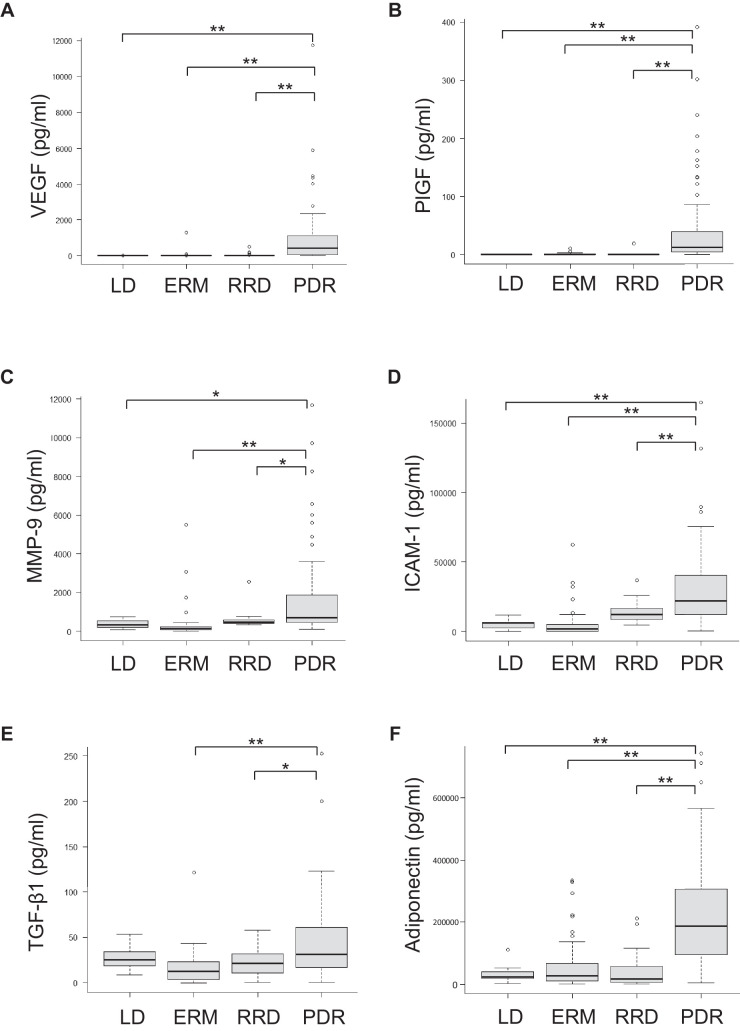
The intravitreal levels of cytokines and adiponectin and correlation between autotaxin (ATX) and these molecules in the vitreous. The intravitreal levels of VEGF (**A**), PlGF (**B**), MMP-9 (**C**), ICAM-1 (**D**), TGF-b (**E**), and adiponectin (**F**) were significantly higher in PDR than those in non-PDR. In patients with PDR, the intravitreal levels of ATX were not significantly correlated with those of vascular endothelial growth factor (VEGF) (**G**), placental growth factor (PlGF) (**H**), or matrix metalloproteinase-9 (MMP-9) (**I**). The intravitreal levels of ATX were significantly correlated with those of intercellular adhesion molecule-1 (ICAM-1) (**J**), transforming growth factor (TGF)-β1 (**K**), and adiponectin (**L**). * P < 0.05, **P < 0.01.

**Figure 3.**
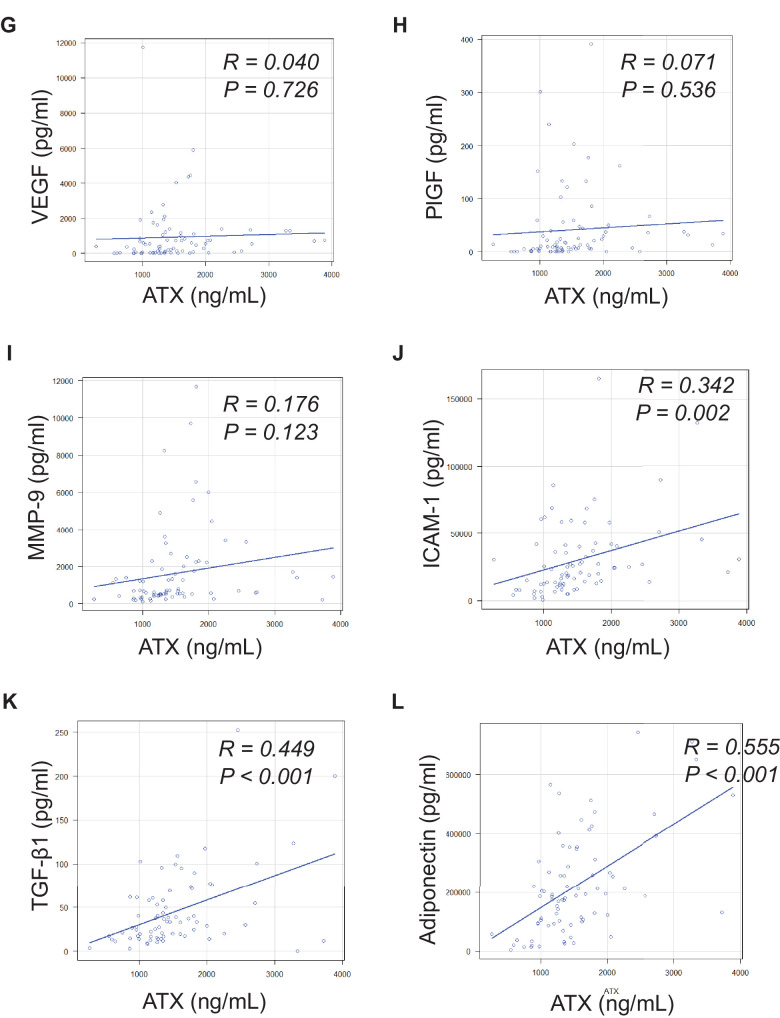
Continued.

### Determination of Antigen for V_A_

**Figure 4.**
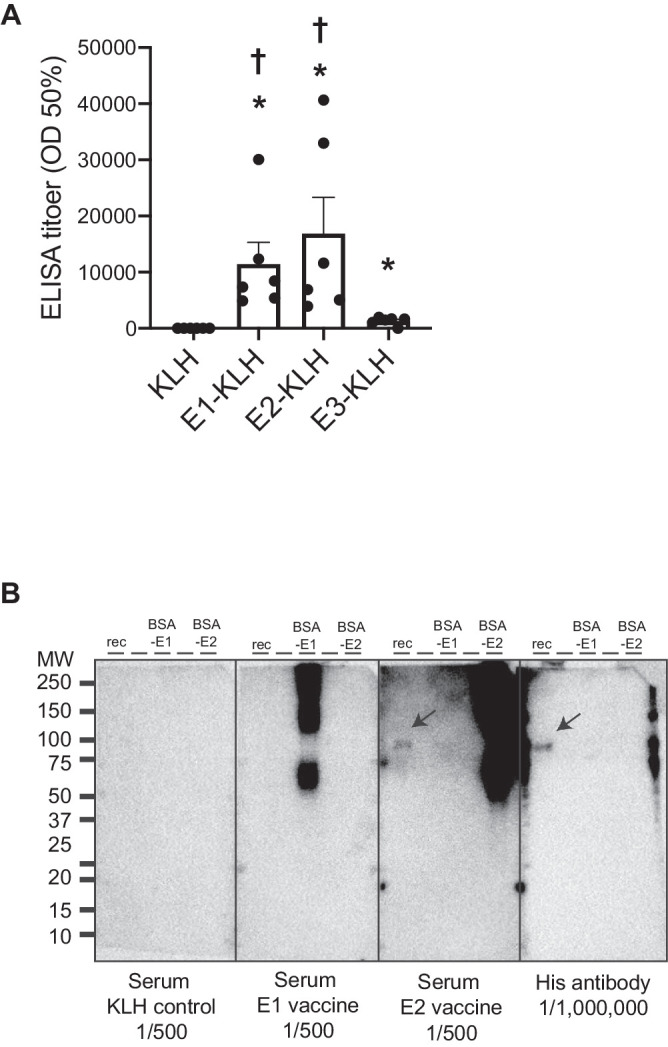
Determination of antigen for autotaxin peptide vaccine. (**A**) Antibody titer at 10 weeks after the first vaccination in C57/BL6 mice. (**B**) Immunoblotting of the serum of immunized C57/BL6 mice. The sera were collected from the mice vaccinated with the keyhole limpet hemocyanin (KLH; control) vaccine, E1 vaccine, and E2 vaccine. His-tag antibody was used as a positive control for his-tag mouse recombinant ATX. *P < 0.05 vs. KLH*, ^†^P* < 0.05 vs. E3-KLH. BSA, bovine serum albumin; KLH, keyhole limpet hemocyanin, rec, recombinant murine autotaxin.

Antibody titers measured at 10 weeks post-immunization were significantly higher in mice that received E1-KLH and E2-KLH injections than those that received KLH (control) and E3-KLH injections. We then tested the specific binding ability of antiserum to recombinant ATX using an immunoblotting assay (Fig. 4B). Significant immunoblotting bands appeared at the molecular weight of ATX in antiserum from the mice that received the E2 injection and antiserum containing a commercially available anti-ATX antibody. Therefore, E2 was determined to be the most suitable antigen for the peptide vaccine.

### Assessment of Systemic and Ocular Parameters in db/db Mice

**Figure 5.**
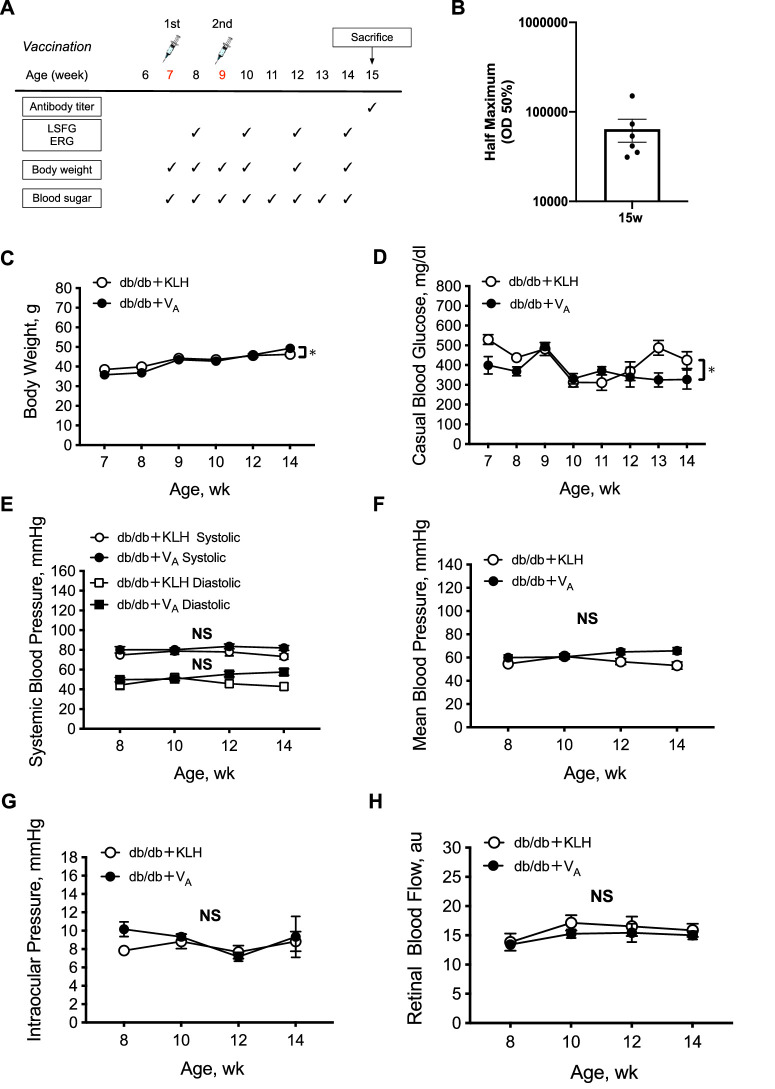
Inoculation of autotaxin peptide vaccine (V_A_) in type 2 diabetic db/db mice. (**A**) The vaccination schedule in db/db mice. (**B**) Antibody titer of V_A_ at 15 weeks of age in db/db mice. (**C–H**) Parameters monitored during the experiment: body weight (**C**), casual blood glucose (**D**), systemic blood pressure (**E**), mean blood pressure (**F**), intraocular pressure (**G**), and retinal blood flow (**H**). *N* = 6 in V_A_ and the KLH group, respectively.

Antibody titers were elevated at 8 weeks post-immunization (15 weeks of age). During the experiment, body weight was significantly heavier in db/db mice treated with V_A_ than in those treated with KLH (control) vaccine (*P* < 0.05). Casual blood glucose was also significantly lower in db/db mice treated with V_A_ than in those treated with the KLH vaccine (*P* < 0.05). No significant differences were found between the two groups in systemic BP, IOP, OPP, or RBF (2-way repeated measures ANOVA; Fig. 5).

### Longitudinal Assessment of Neurovascular Coupling by Measuring RBF Response to Systemic Hyperoxia and Flicker Stimulation in db/db Mice

**Figure 6.**
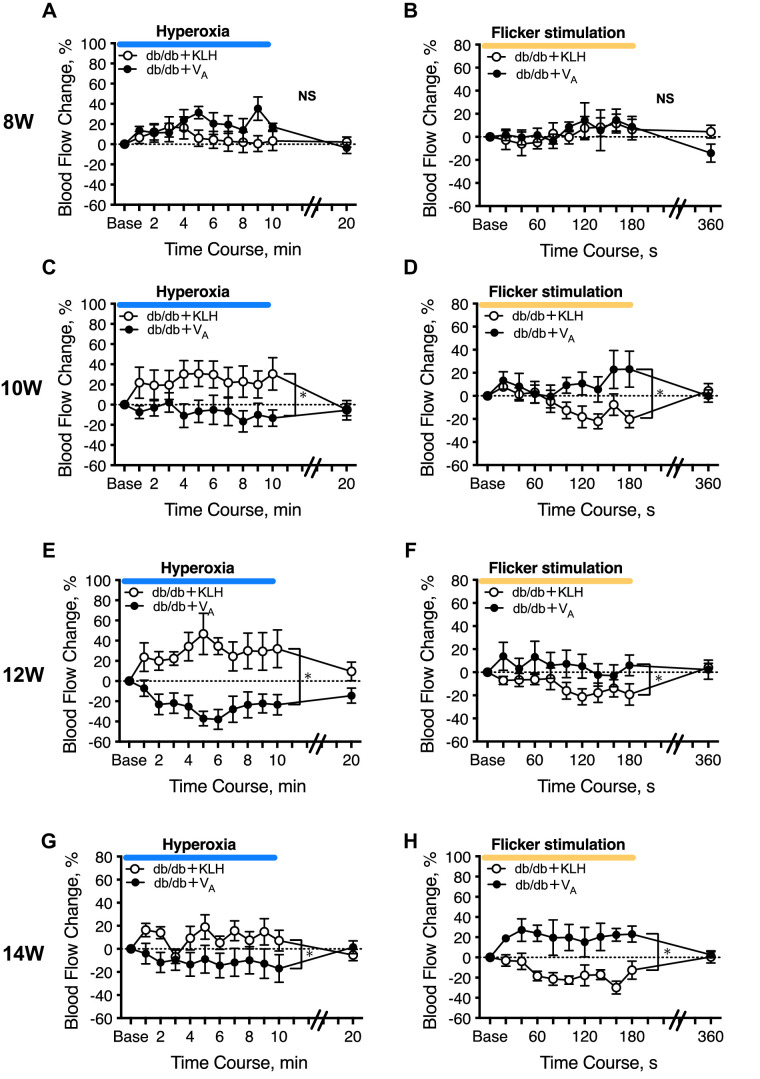
Evaluation of the effect of the autotaxin peptide vaccine (V_A_) on neurovascular coupling in db/db type 2 diabetic mice using laser speckle flowgraphy. (**A, C, E, G**) Time course of systemic hyperoxia-induced blood flow changes in db/db mice at age 8 weeks (**A**), 10 weeks (**C**), 12 weeks (**E**), and 14 weeks (**G**). (**B, D, F, H**) Time course of flicker light-induced blood flow changes in db/db mice at age 8 weeks (**B**), 10 weeks (**D**), 12 weeks (**F**), and 14 weeks (**H**). *N* = 6 in each group. * P < 0.05.

Hyperoxia typically induces a reduction in RBF in non-diabetic mice.^12^ At 8 weeks of age, RBF was not decreased in either group of db/db mice. At 10, 12, and 14 weeks of age, hyperoxia induction led to an increase in RBF in db/db mice treated with KLH, which is consistent with previous reports.^11^^,^^14^ However, db/db mice treated with V_A_ showed hyperoxia-induced reduction of RBF at 10, 12, and 14 weeks of age (Figs. 6A, 6C, 6E, 6G).

Flicker stimulation normally causes a rapid increase in RBF.^12^ At 8 weeks of age, we observed no significant difference in RBF during flicker stimulation. At 10, 12, and 14 weeks of age, we observed a decrease, rather than an increase, in RBF in db/db mice treated with KLH, which is also consistent with previous reports.^11^^,^^14^ In addition, we observed a statistically significant difference in RBF response to flicker stimulation between the KLH and V_A_ groups (Figs. 6B, 6D, 6F, 6H).

### Neuroprotective Effect of V_A_ in db/db Mice

**Figure 7.**
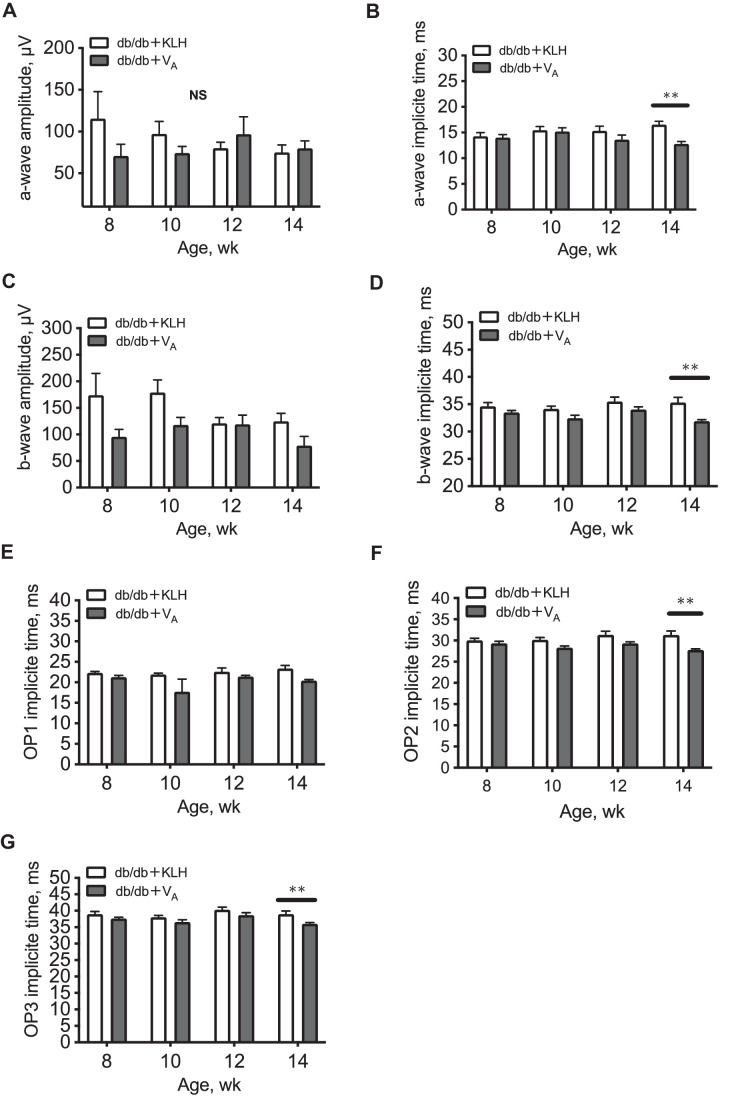
Comparison of neuronal function between keyhole limpet hemocyanin (KLH) (control)- and autotaxin peptide vaccine (V_A_)-treated db/db mice. Analysis included a-wave amplitude (**A**), a-wave implicit time (**B**), b-wave amplitude (**C**), and b-wave implicit time (**D**). Oscillatory potential (OP) was analyzed in components OP1 (**E**), OP2 (**F**), and OP3 (**G**). *N* = 6 in each group. **P < 0.01.

There was no significant difference in the amplitude of a- and b-waves between the KLH and V_A_ groups. However, the implicit times of a- and b-waves were significantly shorter in the V_A_ mice than the KLH mice at 14 weeks of age (*P* < 0.01). To further evaluate the neuronal function of the inner retinal layer, we analyzed the implicit times of OPs. The implicit times of OP2 and OP3 were significantly shorter in the V_A_ mice than the KLH mice at 14 weeks of age (Fig. 7).

## Discussion

The present study attempted to clarify the involvement of ATX in the pathogenesis of DR. For the first time, we demonstrated that ATX was increased, rather than decreased, in the vitreous of patients with PDR. We found that ATX was highly expressed in the retinal endothelial cells of patients with diabetes. Based on this result, we hypothesized that ATX inhibition attenuates the pathogenesis of DR. To test this hypothesis, we developed a novel ATX peptide vaccine and administered it to type 2 diabetic db/db mice. We found that inhibiting ATX activation prevented neuronal dysfunction and neurovascular-coupling impairment in these mice.

A strength of our study is the inclusion of non-diabetic patients with ERM and LD in addition to RRD. The results demonstrated that intravitreal levels of ATX were significantly elevated in PDR and RRD. A previous study that examined intraocular levels of ATX in patients with PDR and RRD concluded that ATX levels were lower in PDR than non-diabetic RRD.^3^ However, that study included only patients with RRD as the non-diabetic controls. RRD is an acute-onset disease characterized by cell proliferation and inflammation that can lead to PVR. In fact, we recently reported that an ATX aptamer reduced the severity of PVR in a porcine model.^15^ Furthermore, the present data also suggest that vitreal ATX levels are higher in RRD than in other non-diabetic eye diseases. Although PDR and RRD are distinct diseases, they share pathological features such as proliferation and fibrosis. Therefore, because this study included non-diabetic retinal diseases other than RRD, our data suggest that ATX levels are higher in PDR than in these non-diabetic diseases.

In the patients with PDR, vitreal levels of LPA and LPC were strongly correlated. LPC is a substrate of LPA, and two active enzymes catalyze this pathway: ATX and acylglycerol kinase. A previous study demonstrated that acylglycerol kinase levels were significantly higher and ATX levels were significantly lower in PDR than RRD.^3^ As mentioned previously, intravitreal injection of an ATX aptamer reduced PVR severity in a porcine model.^15^ This result indicates that intraocular ATX has enzymatic activity and contributes to LPA generation. The present data show a positive correlation between intravitreal ATX and LPA levels, further indicating the involvement of ATX in LPA generation in PDR. Thus, ATX may be partly responsible for LPA production in this disease.

PDR is initiated by the overexpression of hypoxia-induced cytokines, such as VEGF and PlGF.^16^ Abnormal vessel growth typically occurs at the site of vitreous-retinal adhesion, such as the optic disc and blood vessels. Subsequently, abnormal blood vessels continue to grow, accompanied by fibrosis. Our data do not suggest that increased ATX plays a role in the generation of VEGF and PlGF but rather indicate that ATX is involved in the expression of TGF-β1, leading to fibrosis in eyes with PDR. ATX-LPA signaling has been reported to promote angiogenesis by VEGF production.^2^ In addition, LPA stimulation also increases MMP-9 expression in normal human chondrocytes.^17^ In our study, VEGF concentrations in patients with PDR varied widely, possibly due to differences in PDR activity, making it difficult to clarify the relationship between ATX and angiogenic cytokines. However, intravitreal levels of TGF-β1 were elevated and correlated with those of ATX in patients with PDR. Igarashi et al. showed a positive correlation between ATX and TGF-β1 levels in the aqueous humor of patients with Posner-Schlossman syndrome.^18^ The researchers suggested that ATX plays a key role in fibrosis in the trabecular meshwork, leading to higher intraocular pressure. Taken together, these findings suggest that increased ATX levels may contribute to proliferative membrane formation in PDR via TGF-β1 signaling activation.

Adiponectin, an adipocyte-derived hormone, has beneficial anti-inflammatory, anti-oxidative, and vasoprotective effects in patients with obesity and diabetes.^19^ Previous studies reported that intraocular levels of adiponectin were positively correlated with the severity of DR.^20^^–^^22^ Increased adiponectin is considered a compensatory mechanism in DR. Our data also suggest that adiponectin levels were increased in eyes with DR. In addition, our analysis demonstrated for the first time that intraocular levels of adiponectin and ATX were positively correlated in PDR. ATX and adiponectin expression is regulated by the adipocyte microenvironment, but whether ATX and adiponectin are directly associated remains unclear. In addition to adiponectin, leptin is another adipocytokine implicated in the pathogenesis of DR.^23^ In the present study, intravitreal levels of leptin were also higher in PDR than in non-diabetic eye diseases (data not shown), further substantiating the involvement of leptin in the pathogenesis of DR. However, we found no correlation between leptin and ATX expression in PDR (data not shown). Taken together, these findings suggest a potential link between ATX-LPA signaling and adiponectin expression.

ATX inhibitors are currently used for treatment of pulmonary fibrosis.^24^ Oral ATX inhibitors may also be beneficial for DR prevention. However, individuals with diabetes require long-term treatment with these drugs, and treatment efficacy depends heavily on patient adherence. To address this concern, we used a peptide vaccine method to inhibit ATX. In a novel finding, our data suggest that V_A_ improved casual blood glucose control in type 2 diabetes. A previous study demonstrated that an ATX inhibitor reduced blood glucose levels in a type 1 streptozotocin-induced diabetic model.^25^ However, future studies are needed to clarify how ATX inhibition improves blood glucose control in diabetes.

Neurovascular coupling plays a crucial role in maintaining retinal homeostasis.^26^^–^^29^ Impaired neurovascular coupling occurs before significant DR development^14^^,^^28^ and can be observed as early as 10 weeks of age in db/db mice.^14^ In the present study, V_A_ was injected into db/db mice before the occurrence of impaired neurovascular coupling, and we found that V_A_ protected neurovascular coupling from diabetic insults. Müller cells are thought to mediate neurovascular coupling between neurons and blood vessels,^27^ and Müller cell dysfunction is associated with the development of diabetic macular edema.^30^ Although we did not perform immunohistochemical analysis of glial fibrillary acidic protein expression in the retina, we believe that V_A_ therapy may serve as an alternative or adjunctive therapy for diabetic macular edema.

Neuronal dysfunction is also a key feature of early DR.^31^^–^^33^ We previously reported that neuronal dysfunction occurs later than neurovascular coupling dysfunction,^14^ indicating that neuronal cells are not largely responsible for initial impairment of neurovascular coupling. Using ERG, we found that V_A_ improved the implicit time of the waves in this model. Taken together, these results suggest that V_A_ may protect neurons in all retinal layers and Müller glia in type 2 diabetes.

The present study has several limitations. First, we did not assess PDR activity, nor we did measure intraocular ATX levels in non-proliferative DR. Interventions such as anti-VEGF treatment and retinal photocoagulation affect levels of intraocular cytokines, including VEGF, potentially confounding the correlation analysis between ATX and cytokine levels. In addition, the timing of the increase of intraocular ATX levels was not determined. Second, we did not measure LPA levels in the sera and eyes after vaccination. The ATX peptide vaccine's beneficial effects on DR likely depend on intraocular LPA concentrations, meaning that measuring LPA is required to prove the effects of V_A_ in type 2 diabetes. Third, DR is clinically diagnosed based on vascular abnormalities such as microaneurysms, non-perfused areas, and neovascularization. In murine diabetic models, such as STZ and db/db mice, such abnormalities do not develop because of the mice's short lifespan. Therefore, we used the assessment of neurovascular coupling in db/db mice. Although neurovascular dysfunction is one of the earliest pathological changes observed in DR,^14^ it is not specific to this disease and may occur in other retinal disorders. Therefore, our findings should be interpreted with caution, and further studies using DR-specific parameters are warranted. Fourth, we did not determine ATX expression sites in the db/db retinas. An in vitro study previously demonstrated that high glucose stimulation decreased ATX expression and increased VEGF and TGF-β1 expression in cultured retinal pigment epithelium.^34^ To date, however, no studies have identified the precise site of ATX expression in the inner retina under diabetic conditions. Further investigations are therefore required to determine how intraocular ATX increases in diabetes. Last, because the present study focused on investigating the effects of ATX inhibition in diabetes, we did not evaluate how V_A_ affects normal retinal function in non-diabetic controls. Further studies are warranted to facilitate its translation into clinical practice.

In summary, ATX levels were elevated in the eyes of patients with diabetes with PDR. ATX peptide vaccination slightly improved blood glucose control and significantly restored neuronal and glial retinal function in type 2 diabetic mice. These findings suggest that elevated ATX in humans raises the possibility of a contribution to the progression of advanced DR progress, although further studies are needed to clarify its role in PDR. Moreover, additional work is required to determine whether ATX peptide vaccination may serve as an alternative ATX inhibition strategy for the treatment of DR.

## References

[1] Wong TY, Sun J, Kawasaki R, et al. Guidelines on diabetic eye care: the International Council of Ophthalmology Recommendations for Screening, Follow-up, Referral, and Treatment Based on Resource Settings. *Ophthalmology*. 2018; 125(10): 1608–1622. 29776671 10.1016/j.ophtha.2018.04.007

[2] Terao R, Kaneko H. Lipid signaling in ocular neovascularization. *Int J Mol Sci*. 2020; 21(13): 4758. 32635437 10.3390/ijms21134758 PMC7369954

[3] Abu El-Asrar AM, Mohammad G, Nawaz MI, Siddiquei MM, Kangave D, Opdenakker G. Expression of lysophosphatidic acid, autotaxin and acylglycerol kinase as biomarkers in diabetic retinopathy. *Acta Diabetol*. 2013; 50(3): 363–371. 22864860 10.1007/s00592-012-0422-1

[4] Moolenaar WH, Perrakis A. Insights into autotaxin: how to produce and present a lipid mediator. *Nat Rev Mol Cell Biol*. 2011; 12(10): 674–679. 21915140 10.1038/nrm3188

[5] Umezu-Goto M, Kishi Y, Taira A, et al. Autotaxin has lysophospholipase D activity leading to tumor cell growth and motility by lysophosphatidic acid production. *J Cell Biol*. 2002; 158(2): 227–233. 12119361 10.1083/jcb.200204026 PMC2173129

[6] Tokumura A, Majima E, Kariya Y, et al. Identification of human plasma lysophospholipase D, a lysophosphatidic acid-producing enzyme, as autotaxin, a multifunctional phosphodiesterase. *J Biol Chem*. 2002; 277(42): 39436–39442. 12176993 10.1074/jbc.M205623200

[7] Nakagami H, Koriyama H, Morishita R. Peptide vaccines for hypertension and diabetes mellitus. *Vaccines (Basel)*. 2014; 2(4): 832–840. 26344893 10.3390/vaccines2040832 PMC4494253

[8] Takeuchi H, Imamura K, Ji B, et al. Nasal vaccine delivery attenuates brain pathology and cognitive impairment in tauopathy model mice. *NPJ Vaccines*. 2020; 5(1): 28. 32219000 10.1038/s41541-020-0172-y PMC7096417

[9] Tokuhara Y, Kurano M, Shimamoto S, et al. A new enzyme immunoassay for the quantitative determination of classical autotaxins (ATXalpha, ATXbeta, and ATXgamma) and novel autotaxins (ATXdelta and ATXepsilon). *PLoS One*. 2015; 10(6): e0130074. 26083365 10.1371/journal.pone.0130074 PMC4471343

[10] Honjo M, Igarashi N, Kurano M, et al. Autotaxin-lysophosphatidic acid pathway in intraocular pressure regulation and glaucoma subtypes. *Invest Ophthalmol Vis Sci*. 2018; 59(2): 693–701. 29392315 10.1167/iovs.17-23218

[11] Yokota H, Hayashi H, Hanaguri J, et al. Effect of prorenin peptide vaccine on the early phase of diabetic retinopathy in a murine model of type 2 diabetes. *PLoS One*. 2022; 17(1): e0262568. 35041699 10.1371/journal.pone.0262568 PMC8765632

[12] Hanaguri J, Yokota H, Watanabe M, Kuo L, Yamagami S, Nagaoka T. Longitudinal stability of retinal blood flow regulation in response to flicker stimulation and systemic hyperoxia in mice assessed with laser speckle flowgraphy. *Sci Rep*. 2020; 10(1): 19796. 33188259 10.1038/s41598-020-75296-y PMC7666208

[13] Sugiyama T, Araie M, Riva CE, Schmetterer L, Orgul S. Use of laser speckle flowgraphy in ocular blood flow research. *Acta Ophthalmol*. 2010; 88(7): 723–729. 19725814 10.1111/j.1755-3768.2009.01586.x

[14] Hanaguri J, Yokota H, Watanabe M, et al. Retinal blood flow dysregulation precedes neural retinal dysfunction in type 2 diabetic mice. *Sci Rep*. 2021; 11(1): 18401. 34526573 10.1038/s41598-021-97651-3 PMC8443656

[15] Hanazaki H, Yokota H, Yamagami S, Nakamura Y, Nagaoka T. The effect of anti-autotaxin aptamers on the development of proliferative vitreoretinopathy. *Int J Mol Sci*. 2023; 24(21): 15926. 37958909 10.3390/ijms242115926 PMC10647324

[16] Kusuhara S, Fukushima Y, Ogura S, Inoue N, Uemura A. Pathophysiology of diabetic retinopathy: the old and the new. *Diabetes Metab J*. 2018; 42(5): 364–376. 30362302 10.4093/dmj.2018.0182 PMC6202564

[17] Chuang YW, Chang WM, Chen KH, Hong CZ, Chang PJ, Hsu HC. Lysophosphatidic acid enhanced the angiogenic capability of human chondrocytes by regulating Gi/NF-kB-dependent angiogenic factor expression. *PLoS One*. 2014; 9(5): e95180. 24879414 10.1371/journal.pone.0095180 PMC4039431

[18] Igarashi N, Honjo M, Yamagishi R, et al. Involvement of autotaxin in the pathophysiology of elevated intraocular pressure in Posner-Schlossman syndrome. *Sci Rep*. 2020; 10(1): 6265. 32286414 10.1038/s41598-020-63284-1 PMC7156668

[19] Kadowaki T, Yamauchi T, Kubota N, Hara K, Ueki K, Tobe K. Adiponectin and adiponectin receptors in insulin resistance, diabetes, and the metabolic syndrome. *J Clin Invest*. 2006; 116(7): 1784–1792. 16823476 10.1172/JCI29126 PMC1483172

[20] Frystyk J, Tarnow L, Hansen TK, Parving HH, Flyvbjerg A. Increased serum adiponectin levels in type 1 diabetic patients with microvascular complications. *Diabetologia*. 2005; 48(9): 1911–1918. 16078018 10.1007/s00125-005-1850-z

[21] Rodriguez AJ, Nunes Vdos S, Mastronardi CA, Neeman T, Paz-Filho GJ. Association between circulating adipocytokine concentrations and microvascular complications in patients with type 2 diabetes mellitus: a systematic review and meta-analysis of controlled cross-sectional studies. *J Diabetes Complications*. 2016; 30(2): 357–367. 26684169 10.1016/j.jdiacomp.2015.11.004

[22] Yang HS, Choi YJ, Han HY, et al. Serum and aqueous humor adiponectin levels correlate with diabetic retinopathy development and progression. *PLoS One*. 2021; 16(11): e0259683. 34780524 10.1371/journal.pone.0259683 PMC8592425

[23] Gariano RF, Nath AK, D'Amico DJ, Lee T, Sierra-Honigmann MR. Elevation of vitreous leptin in diabetic retinopathy and retinal detachment. *Invest Ophthalmol Vis Sci*. 2000; 41(11): 3576–3581. 11006255

[24] Kato K, Ikeda H, Miyakawa S, et al. Structural basis for specific inhibition of Autotaxin by a DNA aptamer. *Nat Struct Mol Biol*. 2016; 23(5): 395–401. 27043297 10.1038/nsmb.3200

[25] Lee JH, Khin PP, Lee G, Lim OK, Jun HS. Effect of BBT-877, a novel inhibitor of ATX, on a mouse model of type 1 diabetic nephropathy. *Aging (Albany NY)*. 2022; 14(16): 6467–6480. 36036755 10.18632/aging.204249 PMC9467391

[26] Pournaras CJ, Rungger-Brandle E, Riva CE, Hardarson SH, Stefansson E. Regulation of retinal blood flow in health and disease. *Prog Retin Eye Res*. 2008; 27(3): 284–330. 18448380 10.1016/j.preteyeres.2008.02.002

[27] Reichenbach A, Bringmann A. New functions of Muller cells. *Glia*. 2013; 61(5): 651–678. 23440929 10.1002/glia.22477

[28] Garhofer G, Chua J, Tan B, Wong D, Schmidl D, Schmetterer L. Retinal neurovascular coupling in diabetes. *J Clin Med*. 2020; 9(9): 2829. 32882896 10.3390/jcm9092829 PMC7565465

[29] Alarcon-Martinez L, Shiga Y, Villafranca-Baughman D, et al. Neurovascular dysfunction in glaucoma. *Prog Retin Eye Res*. 2023; 97: 101217. 37778617 10.1016/j.preteyeres.2023.101217

[30] Curtis TM, Hamilton R, Yong PH, et al. Muller glial dysfunction during diabetic retinopathy in rats is linked to accumulation of advanced glycation end-products and advanced lipoxidation end-products. *Diabetologia*. 2011; 54(3): 690–698. 21116609 10.1007/s00125-010-1971-x

[31] Kern TS, Barber AJ. Retinal ganglion cells in diabetes. *J Physiol*. 2008; 586(18): 4401–4408. 18565995 10.1113/jphysiol.2008.156695 PMC2614025

[32] Sohn EH, van Dijk HW, Jiao C, et al. Retinal neurodegeneration may precede microvascular changes characteristic of diabetic retinopathy in diabetes mellitus. *Proc Natl Acad Sci USA*. 2016; 113(19): E2655–E2664. 27114552 10.1073/pnas.1522014113 PMC4868487

[33] Vujosevic S, Muraca A, Alkabes M, et al. Early microvascular and neural changes in patients with type 1 and type 2 diabetes mellitus without clinical signs of diabetic retinopathy. *Retina*. 2019; 39(3): 435–445. 29206758 10.1097/IAE.0000000000001990

[34] Liu Y, Yamagishi R, Honjo M, et al. Role of autotaxin in high glucose-induced human ARPE-19 cells. *Int J Mol Sci*. 2022; 23(16): 9181. 36012446 10.3390/ijms23169181 PMC9409272

